# Effects of a Tobacco-Free Work Site Policy on Employee Tobacco Attitudes and Behaviors, Travis County, Texas, 2010–2012

**DOI:** 10.5888/pcd14.170059

**Published:** 2017-12-14

**Authors:** Sarah E. Seidel, Kristi Metzger, Andrea Guerra, Jessie Patton-Levine, Sandeepkumar Singh, William T. Wilson, Philip Huang

**Affiliations:** 1 Austin Public Health, Austin, Texas; 2 Integral Care, Austin, Texas

## Abstract

**Background:**

The adoption of tobacco-free policies in behavioral health settings is an important step in reducing staff tobacco use as well as the high rates of tobacco use among people with mental illness and behavioral disorders. Studies have demonstrated the importance of staff support when implementing tobacco-free workplace policies, but there is limited research examining tobacco use prevalence among staff and staff attitude before and after policy adoption.

**Community Context:**

Integral Care, a local authority for behavioral health and developmental disabilities in Austin, Texas, and Austin Public Health embarked on a comprehensive planning process before implementing a 100% tobacco-free campus policy. The objectives were 1) assess staff tobacco use and attitudes toward a tobacco-free policy, 2) communicate policy to staff, 3) provide staff education and training, and 4) provide cessation resources.

**Methods:**

Integral Care and Austin Public Health conducted a web-based employee survey 6 months before and 6 and 12 months after implementation of the policy to measure tobacco use prevalence and attitudes among employees.

**Outcome:**

Employees had significant improvements in tobacco use prevalence and attitudes toward the tobacco-free policy from pre-implementation to post-implementation. Tobacco use prevalence among staff decreased from 27.6% to 13.8%, and support for the policy increased from 60.6% to 80.3% at 12 months post-implementation.

**Interpretation:**

Adoption of 100% tobacco-free campus policies in behavioral health settings can result in significant reductions in staff tobacco use. Leadership should provide staff with education, training, and cessation support before adoption of tobacco-free work site policies to ensure success.

## Background

Tobacco-free workplace policies can decrease tobacco use among employees (1). The adoption of these policies in health care settings has the potential to reduce tobacco use among staff as well as the patients or clients they serve. Behavioral health care settings present an especially challenging and yet critical setting for the implementation of tobacco-free policies. Smoking rates in individuals with mental illness are 2 to 3 times higher than in the general population (2,3). Health care professionals other than physicians, and specifically health care professionals working long hours, report high rates of tobacco use (4,5). Behavioral health care professionals, in particular, have a smoking prevalence that exceeds that of the general population (6).

While behavioral health care professionals recognize the negative health effects of smoking and the importance of addressing tobacco use among their patients and clients (7,8), the establishment of tobacco-free policies in treatment settings has faced obstacles. Staff cite concerns that tobacco-free policies could negatively impact staff–client relationships (9–12). Organizational barriers include common practices such as promoting patient or client smoking for behavioral reinforcement and staff members and patients smoking together (6,13). Additionally, low levels of staff knowledge, confidence, skills, and perceived responsibility and a lack of training and tobacco use cessation support and resources for patients and staff (6,11) further impede policy adoption and implementation. Staff support is considered crucial to successfully implementing tobacco-free policies in behavioral health treatment settings (14,15). Yet, there is limited research examining 1) the use of participatory methods to address the abovementioned barriers to behavioral health staff support and 2) staff attitudes toward tobacco-free workplace policies and the prevalence of tobacco use among staff before and after policy adoption. We sought to determine whether comprehensive planning before the implementation of a tobacco-free work site policy could decrease employee tobacco use.

## Community Context

In Travis County, Texas, in 2010, 13.4% the population with frequent mental distress were current smokers, compared with 10.7% of the population not reporting mental distress (16). No local data on the prevalence of tobacco use among health care professionals in behavioral health care settings exist, though research suggests that the prevalence of smoking among nonphysician health care professionals, and specifically behavioral health care professionals, remains high (4–6,13).

On February 1, 2011, Integral Care (formerly Austin/Travis County Integral Care), a local authority for behavioral health and developmental disabilities in Austin, adopted a 100% tobacco-free campus policy. This policy prohibited the use of all forms of tobacco and covered all property owned, leased, or used by Integral Care, including indoor and outdoor spaces and common areas, parking lots and driveways (inside and outside personal vehicles), company vehicles, and residential treatment facilities in Travis County. At that time, Integral Care employed about 600 individuals and served about 27,000 consumers at 44 locations each year. Of the 612 staff employed by Integral Care in 2011, 73.2% were female and 26.6% were male, 53.9% were under the age of 40, and 67.7% held a bachelor’s degree or higher.

Before the 2011 policy, Austin Public Health, formerly Austin/Travis County Health and Human Services, engaged staff and administration at Integral Care in a comprehensive planning process comprising assessment, communication, training, and cessation resources. This process was part of a broader community-level effort in Austin and Travis County, under the Communities Putting Prevention to Work initiative from the Centers for Disease Control and Prevention to reduce tobacco use and prevent chronic disease through policy, systems, and environmental change (17). The objectives of this engagement and planning process were to 1) assess staff attitudes toward tobacco-free policies and their implementation; 2) design and promote early and extensive communication before policy implementation; 3) provide staff with training and organizational support to implement and enforce the policy and track patient tobacco use; and 4) provide staff and consumers with cessation resources. Outcomes of interest for the policy implementation were 1) improving staff attitudes toward tobacco-free policies, 2) reducing the prevalence of tobacco use among staff, and 3) reducing the prevalence of tobacco use among consumers (these data are not reported in this evaluation).

## Methods

### Planning and engagement process

Six months before implementation, Integral Care conducted focus groups to inform staff about the impending policy change and to obtain staff input on the components of the implementation process. An online survey (with invitation to participate and survey link sent via employee email) was administered to determine baseline staff tobacco use rates and attitudes toward the upcoming policy change.

Early and extensive communication of the impending policy change to employees, consumers, and partners was an essential component of Integral Care’s implementation process. The Integral Care communication strategy began 5 months prior (Box) to the effective date of the tobacco-free campus policy to gradually introduce the policy, address staff and consumer resistance to the change, and give tobacco users time to prepare for the change. The internal communication strategy, “We Can Quit” (Figure), consisted of positive, nonpunitive messaging via multiple pathways with the goal of educating staff and encouraging tobacco use cessation. Integral Care created a policy change homepage on the employee intranet that included organization and community cessation resources, information on the health consequences of tobacco use, and policy implementation updates. Integral Care also sent organization-wide emails on cessation success stories, policy updates, memoranda, and cessation resources. Brochures and flyers were distributed, and signage was posted at all campus facilities. The external communication strategy comprised memoranda to Integral Care contractors and leased properties regarding the policy change as well as organizational newsletters and reports to partners in Austin/Travis County. Via a link on the Integral Care homepage, the information from the intranet page was available to the public. At the time, the local health department, Austin Public Health, was also implementing an extensive media campaign on the dangers of tobacco use and secondhand smoke exposure. Integral Care held a media launch event with Austin Public Health to celebrate the policy effective date.

**Box. Timeline of Planning and Implementation for Integral Care’s Tobacco-Free Campus Policy**

**Table:** 

Year and Month	Activity
**2010**	
June	Austin Public Health awards Integral Care with subrecipient grant from Communities Putting Prevention to Work
July	Integral Care policy approved by board of trustees
August	Integral Care staff survey (6 months pre-implementation)
September	Internal communication begins (signage, brochures, intranet, cessation resources)
December	External communication begins; signage posted on properties
**2011**	
January	Staff training and education; media event with Austin Public Health
February	Implementation of tobacco-free workplace policy
March	Tobacco Use Assessment (EHR) Implemented
August	Integral Care staff survey (6 months post-implementation)
**2012**	
February	Integral Care staff survey (12 months post-implementation)

**Figure:**
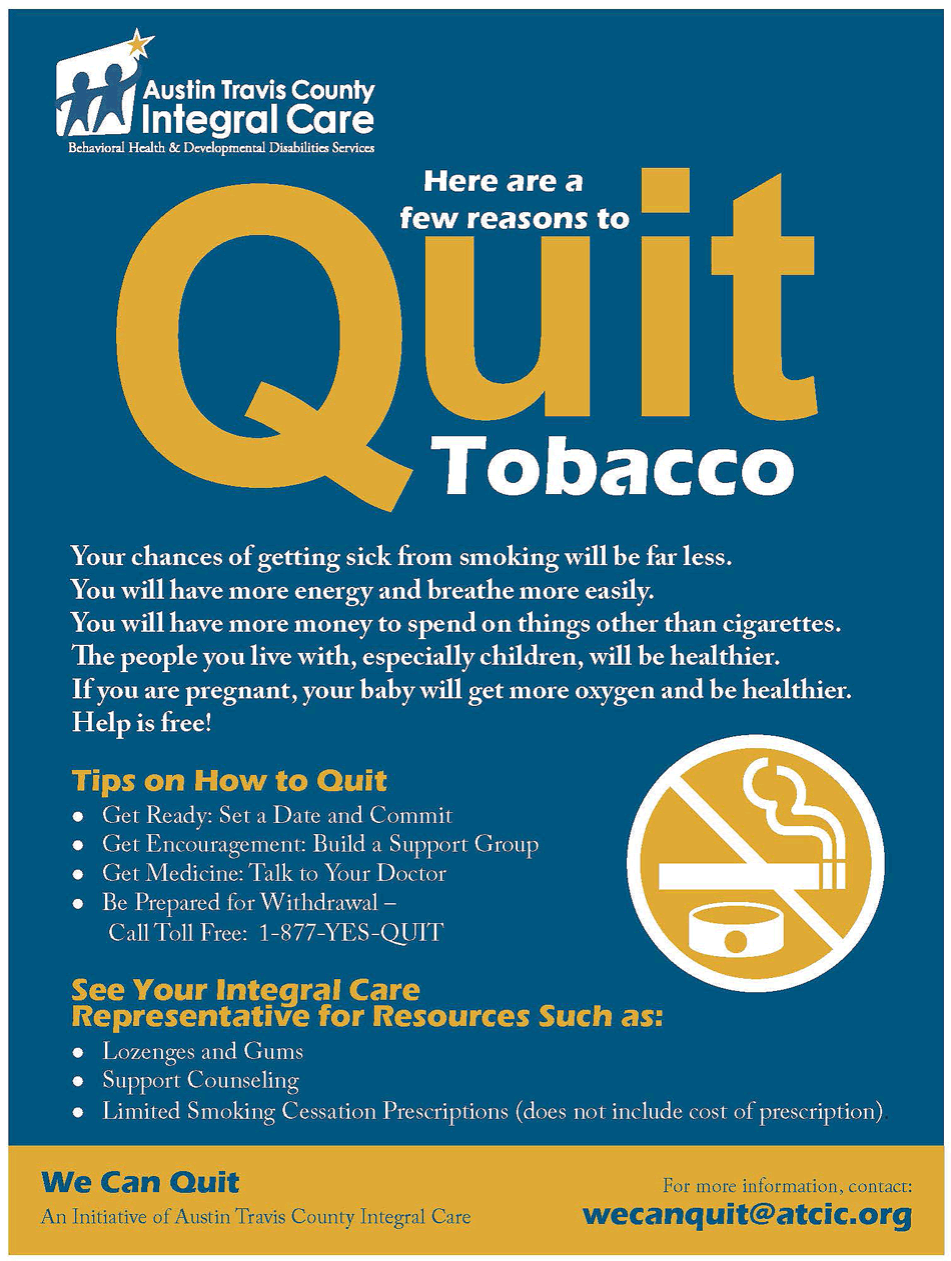
Poster used in the internal communication strategy, We Can Quit, for Integral Care’s tobacco-free campus policy, Austin, Texas, 2010–2012.

Staff training began 90 days before policy implementation (Box) and included education on 1) assessing and treating tobacco use in Integral Care consumers within the clinical setting and 2) how to engage Integral Care staff and consumers about the policy outside the clinical setting. Integral Care implemented the tobacco treatment template from the American Academy of Family Physicians in electronic medical records and trained all clinicians in the Ask and Act treatment protocol (20), pharmacologic treatments, the epidemiology of tobacco use and mental illness or addiction comorbidity, and motivational interviewing techniques. Tobacco use cessation counselors were trained to provide brief (minimum of 15 minutes) counseling sessions for consumers, with up to 6 sessions available to each consumer. Treatment plans and billing reimbursements were adjusted to include tobacco cessation counseling, and staff were trained on accounting for this time. To assist in enforcement, Integral Care staff were also trained on engaging any staff, consumers, and visitors not complying with the policy using brief, nonconfrontational, scripted messages.

During the 6 months before policy implementation (Box), Integral Care also began a comprehensive education and cessation support program for both employees and consumers. Cessation support included free cessation counseling through the Employee Assistance Program and nicotine replacement therapy for both staff and consumers. Additionally, staff were eligible for reimbursement of their first tobacco cessation medical office visit and Zyban (GlaxoSmithKline; tobacco cessation prescription medicine) at no cost. Community cessation resources (eg, the Texas Quit Line) were also promoted by Integral Care.

### Design and analysis

In August and September 2010, 6 months before policy implementation, an 18-question web-based pre-implementation survey was administered to all clinical and nonclinical employees at Integral Care; employees were sent an email with a hyperlink to the survey. The pre-implementation survey had 7 demographic and health questions and 11 questions regarding tobacco use, policy attitudes, and preferred cessation resources. The survey (including additional questions) was administered again at 6 and 12 months post-implementation. Pre- and post-implementation surveys asked Integral Care employees about current tobacco use (ever and while at work), knowledge about the health effects of tobacco use and secondhand smoke, desire to quit, preferred cessation methods, policy support, and willingness to enforce the policy. Response categories for policy support and for willingness to enforce the policy were collapsed from yes, no, and maybe into yes and no/maybe. The post-implementation surveys included 8 additional questions on previous quit attempts, awareness and use of cessation resources, and attitudes about training, enforcement, and compliance. Survey questions relating to the pre-implementation policy environment were modified in the post-implementation surveys to reflect the change in conditions. For example, the pre-implementation survey question “Would you support a tobacco-free policy?” was changed to “Do you support a tobacco-free policy?” The question “Would be willing to assist in the enforcement of the tobacco-free workplace policy?” was changed to “Do you assist in enforcing the tobacco-free workplace policy?”

Pre- and post-implementation survey respondents were not matched, but the only significant difference among the samples in the 3 surveys in terms of sex, age, education, or position was the proportion of staff with a high school diploma or general equivalency diploma as their highest level of education (pre-implementation vs 12 months post-implementation) (Table 1). In each survey, roughly half of respondents were younger than 40 years of age, and approximately three-quarters of respondents were women. The percentage of respondents with a bachelor’s degree or higher was 76% or higher across the 3 surveys. Additionally, survey participants’ demographic characteristics (sex, age, and education) were compared with demographics of the entire Integral Care staff (obtained from de-identified employee data). Race/ethnicity of employees was not provided and was not collected in the survey. No significant differences in sex, age, or education were observed between the survey participants and the study population.

**Table 1:** Characteristics of Surveyed Staff at 6 Months Before and 6 and 12 Months After Implementation of a Tobacco-Free Workplace Policy at Integral Care, Austin, Texas, 2010–2012

Characteristic	6 Months Before Policy Implementation (July–August 2010), n (%)	6 Months After Policy Implementation (July–August 2011), n (%)	12 Months After Policy Implementation (February–April 2012), n (%)
**Total**	246 (100)	209 (100)	222 (100)
**Sex**			
Male	58 (23.6)	49 (23.4)	58 (26.1)
Female	188 (76.4)	160 (76.6)	164 (73.9)
**Age**			
≤30	60 (24.4)	48 (23.0)	54 (24.3)
31–40	61 (24.8)	61 (29.2)	63 (28.4)
41–50	55 (22.4)	43 (20.6)	46 (20.7)
51–60	48 (19.5)	37 (17.7)	43 (19.4)
≥60	22 (8.9)	20 (9.6)	16 (7.2)
**Position at Integral Care^a^**			
Administration	108 (43.9)	99 (47.4)	86 (38.7)
Allied health professional	25 (10.2)	24 (11.5)	26 (11.7)
Direct care staff	83 (33.7)	69 (33.0)	90 (40.5)
Nursing	9 (3.7)	5 (2.4)	2 (0.9)
Physician	3 (1.2)	3 (1.4)	3 (1.4)
Other	18 (7.3)	9 (4.3)	15 (6.8)
**Education**			
High school or general equivalency diploma	34 (13.8^b^)	19 (9.1)	12 (5.4)^b^
Associate’s degree	24 (9.8)	16 (7.7)	25 (11.3)
Bachelor’s degree	73 (29.7)	70 (33.5)	70 (31.5)
Master’s degree	95 (38.6)	87 (37.8)	97 (41.5)
MD or PhD degree	8 (3.3)	5 (2.4)	7 (3.2)
Other	12 (4.9)	12 (5.7)	11 (5.0)

a Job/position categories were chosen to determine employee involvement with Integral Care consumers and do not reflect education levels.

b χ^2^ = 9.32, df = 1; *P* = .002.

Pearson χ^2^ tests were performed to compare survey samples and to compare pre- and post-implementation survey data on tobacco use prevalence, attitudes toward tobacco use, and tobacco-free workplace policy support and to compare tobacco use prevalence change for sex, age, education, and position subgroups. Significance was set at α = .01. Statistical analysis of survey data was performed using Stata 14.0 (StataCorp LP). Research was approved by Integral Care’s Board of Trustees and by the Integral Care Institutional Review Board.

## Outcome

Of approximately 612 eligible Integral Care staff, 246 employees completed the pre-implementation surveys in August and September 2010; 230 employees completed a post-implementation survey at 6 months in July and August 2011 and 234 employees completed a post-implementation survey at 12 months in February through April 2012 (Table 1). Demographic characteristics were missing for 21 respondents in the 6-month post-implementation survey and 12 respondents in the 12-month post-implementation survey. The response rate for the pre-implementation survey was 40.2%, for the 6 months post-implementation survey was 37.6%, and for the 12 months post-implementation survey was 38.2%. 

Tobacco use among staff declined significantly from 27.6% in the pre-implementation survey to 11.6% in the 6-month post-implementation survey (χ^2^ = 18.47; *P* < .001) and to 13.8% in the 12-month post-implementation survey (χ^2^ = 13.43; *P* < .001) (Table 2). There was no significant change in tobacco-use prevalence between the 6- and 12-month post-implementation surveys.

**Table 2:** Changes in Tobacco Use and Support for a Tobacco-Free Campus Policy at 6 Months Before and 6 and 12 Months After Implementation, Integral Care, Austin, Texas, 2010–2012

Characteristic	6 Months Before (July–August 2010) (N = 246)		6 Months After (July–August 2011) (N = 216)		12 Months After (February–April 2012) (N = 224)		*P* Value		
	No. of Respondents	% (n)	No. of Respondents	% (n)	No. of Respondents	% (n)	2010 vs 2011	2010 vs 2012	2011 vs 2012
**Tobacco use prevalence**	246	27.6 (68)	216	11.6 (25)	224	13.8 (31)	<.001	<.001	.48^a^
**Supports tobacco-free workplace^b^**	246	60.6 (149)	216	71.8 (155)	224	83.9 (188)	.11	<.001	.002
Support among tobacco users	68	26.5 (18)	25	60.0 (15)	31	64.5 (20)	.003	<.001	.73
Support among non–tobacco users	178	73.6 (131)	191	72.8 (139)	193	87.0 (168)	.86^c^	.001	<.001
**Assists in enforcement of tobacco-free workplace policy^d^**	246	48.0 (118)	216	61.6 (133)	224	66.1 (148)	.003	<.001	.33
Willingness among tobacco users	68	26.5 (18)	25	64.0 (16)	31	54.8 (17)	.001	.006	.49^c^
Willingness among non–tobacco users	178	56.2 (100)	191	60.7 (116)	193	67.9 (131)	.38	.02	.14

a Nonsignificant increase in proportion observed.

b The survey 6 months before implementation asked, “Would you support a tobacco-free policy?” The surveys after implementation asked, “Do you support a tobacco-free policy?”

c Nonsignificant decrease in proportion observed.

d The survey 6 months before implementation asked, “Would be willing to assist in the enforcement of the tobacco-free workplace policy?” The surveys after implementation asked, “Do you assist in enforcing the tobacco-free workplace policy?”

Staff support for the tobacco-free campus policy increased significantly from 60.6% to 83.9% from pre-implementation to 12 months post-implementation (Table 2) (χ^2^ = 31.53; *P* < .001). Policy support also increased significantly between the 6-month post-implementation (71.8%) and the 12-month post-implementation surveys (χ^2^ = 9.48; *P* = .002). Among tobacco users, there was a significant increase in support of the policy from the pre-implementation survey (26.5%) to the 6-month post-implementation survey (60.0%; χ^2^ = 8.98; *P* = .003) and to the 12-month post-implementation survey (64.5%; χ^2^ = 13.03; *P* < .001). Among non–tobacco users, support for the policy decreased slightly between pre-implementation and 6 months post-implementation. Yet, support increased significantly between the 6-month (72.8%) and 12-month (87.0%) post-implementation surveys (χ^2^ = 12.20; *P* < .001). In all 3 surveys a higher percentage of staff who were non–tobacco users supported the policy than staff who used tobacco.

Most respondents were aware of the cessation services provided by Integral Care in both the 6-month (185; 80.4%) and 12-month (206; 88.0%) post-implementation surveys; tobacco users specifically reported only marginally higher awareness of resources in both post-implementation surveys. Cessation services were not offered to Integral Care staff at the time of the pre-implementation survey, and thus the question was not included in the pre-implementation survey.

In addition, 73% of respondents in the 6-month post-implementation survey and 69% in the 12-month post-implementation survey reported that they felt adequately trained or competent to engage consumers about the tobacco-free policy. There was a significant increase in the percentage of respondents who reported currently assisting in enforcement at 6 months (61.6%) and 12 months post-implementation (66.1%) compared with respondents who reported that they would be willing to assist in enforcement of the policy in the pre-implementation survey (48.0%) (Table 2). Among tobacco users specifically, this significant increase was observed between pre-implementation survey (26.5%) and 6 months post-implementation (64.0%) as well as between pre-implementation and 12 months post-implementation (54.8%). There was a small but nonsignificant decrease in the proportion of tobacco users willing to enforce the policy between 6 and 12 months post-implementation.

In the pre-, 6-month post-, and 12-month post-implementation surveys, 71.7%, 84.0%, and 74.2%, respectively, of tobacco users responded yes or maybe to the question of whether they wanted to quit using tobacco. Of tobacco users, 64.2%, 72.0%, and 64.5%, respectively, responded yes or maybe to the question of whether they were seriously considering quitting in the next 6 months. Approximately half of all tobacco users in the 6-month post-implementation and 12-month post-implementation surveys (56.0% and 45.2%, respectively) had made a quit attempt in the past 9 months (the question was not asked in the pre-implementation survey).

## Interpretation

This study evaluated the effects of a 100% tobacco-free campus policy at a large multisite provider of behavioral health and developmental disabilities services on staff tobacco use rates and staff attitudes toward a tobacco-free campus policy. The objectives of this engagement and planning process (assessment, communication, training, and cessation resources) were all met during the 18-month period. Assessment using web-based surveys of employees was carried out at 6 months before and 6 and 12 months following policy implementation. Regarding communication, survey results indicated that nearly 70% of respondents at both 6 and 12 months post-implementation felt adequately trained in engaging consumers regarding the policy; over 80% of respondents (and over 90% of tobacco users specifically) in both post-implementation surveys were aware of cessation resources. Outcomes of interest for the policy implementation also demonstrated improvement. Staff attitudes in support of tobacco-free policies increased significantly, and staff tobacco use prevalence decreased. Across all 3 surveys there was a high percentage of tobacco users (>64%) intending to quit tobacco in the next 6 months.

This evaluation demonstrates that a comprehensive implementation plan combining education, communication, and cessation support for staff before a tobacco-free policy adoption can contribute to reduced staff tobacco use and increased support for the policy after adoption. The components of education and training for staff regarding smoking behaviors and risks and smoking cessation treatment options for clinical populations have been recommended and linked to the success of smoke-free initiatives in inpatient mental health facilities (15,18). To our knowledge this is the first study of a policy implementation that has incorporated components to address staff tobacco use before implementing the policy in the patient population. Staff commonly cite low or lack of support for tobacco-free policies from the organization in which they work (12,19). Thus, addressing staff needs is an important first step to successfully implementing tobacco-free policies in behavioral health services settings.

Changes that were not significant at 6 months post-implementation (eg, support for the policy among non–tobacco users) were significant at 12 months post-implementation. This indicates that attitudes toward a tobacco-free policy can continue to improve after implementation and suggests that attitudes (and possibly social norms) may not change until after a policy is implemented and individuals can observe consequences or implications of the change.

This study has several limitations. Integral Care provides developmental disabilities services in addition to behavioral health services. Staff providing these services made up 18% of full-time employees in 2011 and may be different than staff working in traditional behavioral health care settings. Additionally, we did not obtain information on staff turnover. Staff may have not received the full intervention if they left or were hired during the 6 months before implementation during which communication, education, and training were conducted. Though survey response rates were average, staff who answered the surveys may have been systematically different than those who did not participate and may not represent the characteristics and attitudes of the Integral Care study population. We were also unable to link pre-implementation and post-implementation survey respondents. However, the only significant difference in demographic characteristics among the pre-implementation and post-implementation survey samples was in the proportion of staff with a high school diploma or general equivalency diploma as their highest level of education (pre-implementation vs 12 months post-implementation). There were no significant differences in the demographic characteristics between any pre-implementation or post-implementation survey samples and the entire Integral Care employee population. However, because employee race/ethnicity data were either not available or not collected, and disparities in tobacco use exist among racial/ethnic groups in the general population, we cannot determine the contribution of race/ethnicity to study findings. Additionally, tobacco users who quit may have been more willing to answer the post-implementation surveys than those who did not quit.

Finally, Integral Care was a sub-recipient of the Communities Putting Prevention to Work grant received by Austin Public Health in 2010. The grant funds provided Integral Care with funding for personnel, operating expenses (including signage), and indirect expenses to plan and execute the policy change. An intervention of this scale may not be feasible for smaller behavioral health providers.

## References

[1] Osinubi OY , Sinha S , Rovner E , Perez-Lugo M , Jain NJ , Demissie K , Efficacy of tobacco dependence treatment in the context of a “smoke-free grounds” worksite policy: a case study. Am J Ind Med 2004;46(2):180–7. 10.1002/ajim.20020 15273971

[2] Lawrence D , Mitrou F , Zubrick SR . Smoking and mental illness: results from population surveys in Australia and the United States. BMC Public Health 2009;9(1):285. 10.1186/1471-2458-9-285 19664203 PMC2734850

[3] McClave AK , McKnight-Eily LR , Davis SP , Dube SR . Smoking characteristics of adults with selected lifetime mental illnesses: results from the 2007 National Health Interview Survey. Am J Public Health 2010;100(12):2464–72. 10.2105/AJPH.2009.188136 20966369 PMC2978196

[4] Shahbazi S , Arif AA , Portwood SG , Thompson ME . Risk factors of smoking among health care professionals. J Prim Care Community Health 2014;5(4):228–33. 10.1177/2150131914527618 24695770

[5] Centers for Disease Control and Prevention. Current cigarette smoking prevalence among working adults — United States, 2004–2010. MMWR Morb Mortal Wkly Rep 2011;60(38):1305–9. 21956406

[6] Johnson JL , Malchy LA , Ratner PA , Hossain S , Procyshyn RM , Bottorff JL , Community mental healthcare providers’ attitudes and practices related to smoking cessation interventions for people living with severe mental illness. Patient Educ Couns 2009;77(2):289–95. 10.1016/j.pec.2009.02.013 19398293

[7] Schroeder SA , Morris CD . Confronting a neglected epidemic: tobacco cessation for persons with mental illnesses and substance abuse problems. Annu Rev Public Health 2010;31(1):297–314, 1p, 314. 10.1146/annurev.publhealth.012809.103701 20001818

[8] Naegle M , Baird C , Farchaus Stein K . Psychiatric nurses as champions for smoking cessation. J Am Psychiatr Nurses Assoc 2009;15(1):21–3. 10.1177/1078390308331092 21665791

[9] Parker C , McNeill A , Ratschen E . Tailored tobacco dependence support for mental health patients: a model for inpatient and community services. Addiction 2012;107(Suppl 2):18–25. 10.1111/j.1360-0443.2012.04082.x 23121356

[10] Hollen V , Ortiz G , Schacht L , Mojarrad MG , Lane GM Jr , Parks JJ . Effects of adopting a smoke-free policy in state psychiatric hospitals. Psychiatr Serv 2010;61(9):899–904. 10.1176/ps.2010.61.9.899 20810588

[11] Ratschen E , Britton J , Doody GA , Leonardi-Bee J , McNeill A . Tobacco dependence, treatment and smoke-free policies: a survey of mental health professionals’ knowledge and attitudes. Gen Hosp Psychiatry 2009;31(6):576–82. 10.1016/j.genhosppsych.2009.08.003 19892217

[12] Hehir AM , Indig D , Prosser S , Archer VA . Implementation of a smoke-free policy in a high secure mental health inpatient facility: staff survey to describe experience and attitudes. BMC Public Health 2013;13(1):315. 10.1186/1471-2458-13-315 23566256 PMC3648483

[13] Dickens GL , Stubbs JH , Haw CM . Smoking and mental health nurses: a survey of clinical staff in a psychiatric hospital. J Psychiatr Ment Health Nurs 2004;11(4):445–51. 10.1111/j.1365-2850.2004.00741.x 15255919

[14] Campion J , Checinski K , Nurse J , McNeill A . Smoking by people with mental illness and benefits of smoke-free mental health services. Adv Psychiatr Treat 2008;14(3):217–28. 10.1192/apt.bp.108.005710

[15] Lawn S , Campion J . Factors associated with success of smoke-free initiatives in Australian psychiatric inpatient units. Psychiatr Serv 2010;61(3):300–5. 10.1176/ps.2010.61.3.300 20194408

[16] Centers for Disease Control and Prevention. 2010 Behavioral Risk Factor Surveillance System questionnaire. https://www.cdc.gov/brfss/questionnaires/pdf-ques/2010brfss.pdf. Accessed October 18, 2017.

[17] Bunnell R , O’Neil D , Soler R , Payne R , Giles WH , Collins J , Fifty communities putting prevention to work: accelerating chronic disease prevention through policy, systems and environmental change. J Community Health 2012;37(5):1081–90. 10.1007/s10900-012-9542-3 22323099

[18] Magor-Blatch LE , Rugendyke AR . Going smoke-free: attitudes of mental health professionals to policy change. J Psychiatr Ment Health Nurs 2016;23(5):290–302. 10.1111/jpm.12309 27278902

[19] Bloor RN , Meeson L , Crome IB . The effects of a non-smoking policy on nursing staff smoking behaviour and attitudes in a psychiatric hospital. J Psychiatr Ment Health Nurs 2006;13(2):188–96. 10.1111/j.1365-2850.2006.00940.x 16608474

[20] American Academy of Physicians. Ask and Act. http://www.aafp.org/about/initiatives/ask-act.html. Accessed October 18, 2017.

